# An integrative bioinformatic predictor of protein sub-cellular localisation in malaria

**DOI:** 10.1186/1471-2105-12-S11-A6

**Published:** 2011-11-21

**Authors:** Ben J Woodcroft, Robert Radloff, Lee M Yeoh, Kristie-Lee Scanlon, Maria A Doyle, Giel G van Dooren, Geoffrey I McFadden, Christopher J Tonkin, Terence P Speed, Stuart A Ralph

**Affiliations:** 1 Department of Biochemistry & Molecular Biology, Bio21 Molecular Science and Biotechnology Institute, University of Melbourne, Australia; 2 The Walter and Eliza Hall Institute of Medical Research, Melbourne, Australia; 3 Institute for Biochemistry, University of Stuttgart, Stuttgart, Germany; 4 Plant Cell Biology Research Centre, School of Botany, University of Melbourne, Melbourne, Australia; 5 Bioinformatics Core Facility, Peter MacCallum Cancer Centre, Melbourne, Australia

## Background

The malarial parasite *Plasmodium falciparum* remains a leading international cause of mortality, with almost a million deaths each year. Determination of protein sub-cellular localisation remains a challenge in *Plasmodium *parasites due to their evolutionary distance from well-studied model organisms, and limited efficiency of appropriate molecular tools. However, abundant large scale systems biology information exist for several *Plasmodium *species as well as other apicomplexan parasites, including full genomic DNA sequences, plus data sets relating to the transcriptome, protein expression and interactions, polymorphisms and phyletic profiles. To date, most bioinformatic predictors of sub-cellular localisation use sequence information exclusively without consideration for other data sets.

## Materials and methods

We developed the first global bioinformatic predictor of sub-cellular localisation in *Plasmodium falciparum* (called Plasmarithm) that predicts localisation for multiple cellular compartments using a variety of post-genomic information types.

## Results and conclusions

**Figure 1:**
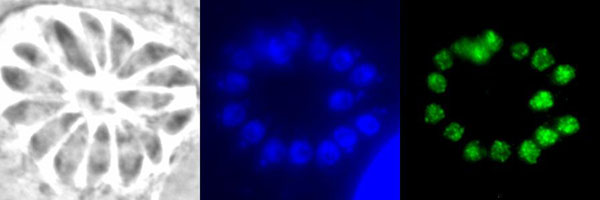
Experimental confirmation of a Plasmarithm localisation prediction. A *P. falciparum* protein (PlasmoDB ID PFE0425w) was predicted to be nuclear, and here the HA tagged *T. gondii* orthologue (ToxoDB ID TGME49_044840) is shown exhibiting a nuclear localisation. The panels show bright field, Hoechst (nuclear) stained, and HA tagged TGME49_044840 images, respectively.

We identified several non-sequence data types that are predictive of localisation, including phyletic distribution and transcript abundance at specific life stages. We performed a comprehensive literature survey of the phylum Apicomplexa to construct a database of >850 recorded protein localisations curated from >700 separate publications. The database, called ApiLoc (freely available at http://apiloc.bio21.unimelb.edu.au), was used to improve the accuracy of our predictor. We achieved an overall accuracy of ~60% on a seven class problem, where a number of the classes have not previously been predicted. To further validate these in-silico analyses, we have experimentally verified localisations of a number of hypothetical proteins in the related apicomplexan *Toxoplasma gondii* (Figure 1).

